# Retinal vascular changes and arterial stiffness during 8-month isolation and confinement: the SIRIUS-21 space analog mission

**DOI:** 10.3389/fphys.2024.1374309

**Published:** 2024-05-27

**Authors:** Adel B. Elmoselhi, Vishwajeet Shankhwar, Rizwan Qaisar, Rifat Hamoudi, Bianca Brix, Adam Salon, Nandu Goswami

**Affiliations:** ^1^ College of Medicine, University of Sharjah, Sharjah, United Arab Emirates; ^2^ Research Institute for Medical and Health Sciences, University of Sharjah, Sharjah, United Arab Emirates; ^3^ Mohammed Bin Rashid University of Medicine and Health Sciences (MBRU), Dubai, United Arab Emirates; ^4^ Division of Surgery and Interventional Science, University College London, London, United Kingdom; ^5^ Division of Physiology and Pathophysiology, Otto Loewi Research Center for Vascular Biology, Immunology, and Inflammation, Medical University of Graz, Graz, Austria; ^6^ Faculty of Health and Social Sciences, Inland Norway University of Applied Sciences, Lillehammer, Norway

**Keywords:** isolation and confinement, cardiovascular and cerebrovascular disorders, SIRIUS-21, space flight, retinal vasculature, analog mission

## Abstract

**Introduction:**

Isolation and confinement are significant stressors during space travel that can impact crewmembers’ physical and mental health. Space travel has been shown to accelerate vascular aging and increase the risk of cardiovascular and cerebrovascular disorders. However, the effect of prolonged isolation and confinement on microvascular function has not yet been thoroughly investigated.

**Methods:**

Retinal vascular imaging was conducted on four crewmembers during- and post-8-month SIRIUS-21 space analog mission. Central retinal arteriolar equivalent (CRAE), central retinal venular equivalent (CRVE), and arteriovenous ratio (AVR) were measured. Pulse wave velocity (PWV), an indicator of arterial stiffness, was also measured.

**Results:**

Data from 4 participants was analyzed. These participants had a mean age of 34.75 ± 5.44 years, height of 170.00 ± 2.00 cm, weight of 74.50 ± 12.53 kg, and average BMI of 25.47 ± 3.94 kg/m^2^. During- and post-isolation, average CRVE showed an upward trend (Pearson’s r 0.784, R-square 0.62), suggesting a dilation of retinal venules, while AVR showed a downward trend (Pearson’s r −0.238, R-square 0.057), which is suggestive of a higher risk of cardiovascular and cerebrovascular dysfunctions. But neither of these trends were statistically significant. Additionally, the average PWV showed an upward trend during- and after-isolation across all crew members.

**Conclusion:**

Isolation and confinement appear to contribute towards retinal vascular damage and arterial stiffness. This cautiously suggests an increased risk of cardiovascular and cerebrovascular disorders due to the contribution of the isolation in space flight. Further studies are needed to confirm and expand on these results as we prepare for future manned missions to the Moon and Mars.

## Introduction

Space travel exposes crew members to various stressors such as microgravity, radiation as well as isolation and confinement. Consequently, there are multiple deleterious effects of these stressors on several organs systems of the space travelers, including cardiovascular and cerebrovascular disorders. However, the causes and mechanisms underlying those disorders in isolation, confined and extreme environments (ICE) are not fully understood. To dissect out the effect of these stressors on vascular health, we studied the effect of long-term isolation and confinement on the changes of retinal blood vessels and arterial stiffness, during- and post-8-month Scientific International Research in Unique terrestrial Station (SIRIUS) analog mission.

Isolation and confinement, in general, are inherent stressors that can significantly perturb human physiology, as they are associated with increased activation of stress pathways (Pagel and Chouker, 2016). Among various physiological systems, the human vasculature is particularly vulnerable to the effects of neuronal and hormonal stress (Schakman et al., 2013; Cacioppo et al., 2015). SIRIUS-21 space analog mission offered an excellent opportunity to investigate the impact of these stressors on human vasculature in a controlled environment, which mimicked the isolation of spaceflight. Previous studies have demonstrated that confinement and isolation during the MARS-500 mission resulted in pathological structural changes in peripheral vasculature (Arbeille et al., 2014). However, the effects of isolation and confinement on microvascular functions and arterial stiffness in long-duration missions have not been fully explored.

The purpose of this study is to investigate the changes in microvascular functions and arterial stiffness that occur during an 8-month SIRIUS analog mission and how long it took the variables to return to baseline values following the isolation. We used non-invasive clinical diagnostic methods such as the Pulse Wave Velocity (PWV) to assess vascular stiffness and measured the retinal microvasculature changes, including the Central Retinal Arteriolar Equivalent (CRAE), the Central Retinal Venular Equivalent (CRVE), and the arteriovenous ratio (AVR) during- and after-isolation. The vascular retinal measurements reflect cardiovascular and cerebrovascular microvasculature beds. In particular, decreases in AVR predict development of cerebral atrophy, stroke and other cardiovascular events in adults (French et al., 2022).

The findings of this study help us better understand the effects of ICE on microvasculature. This research is important as we plan for ambitious manned missions to the Moon and Mars. Similarly, the knowledge gained from such studies can be important in developing effective countermeasures to mitigate negative effects of isolation and confinement on human physiology.

## Materials and methods

### Study design and population

The study was conducted jointly by Roscosmos, NASA, and Mohammed Bin Rashid Space Centre (MBRSC), Dubai at the refurbished isolation facility located at the Institute of Biomedical Problems (IBMP) in Moscow, Russia between 4 November 2021, until 3 July 2022. The current study was one of multiple projects in an international large study named SIRIUS-21. The investigation of the effects of 8-month of confinement initially started with a group of six crewmembers. Following 33 days from the beginning of the mission, one of the crewmembers withdrew because of an unexpected health issue following a minor arm injury during exercise. However, the mission was completed with the remaining crew (three men and two women) including Russian, American and Arab background participants. The recruitment and the inclusion and exclusion criteria with healthy young and middle age healthy individuals, which were selected by IBMP leadership scientific team, in collaboration with NASA and MBRSC. During the 8-month confinement period, the crew members resided in a cylindrical module with a volume of 500 m^3^, engaging in experimental scientific activities that involved moderate physical exertion (Schneider et al., 2013). They lived in conditions similar to those on Earth, though within a restricted space. Throughout the duration, the subjects maintained limited yet regular contact with the control center. Out of the nine measurement time points conducted during the mission (DM), seven were found reliable and analyzed on days 68–69 (DM3), day 93–95 (DM 4), day 109–111 (DM 5), day 154–156 (DM6), day 180–182 (DM7), day 222–224 (DM8), day 238–239 (DM9). Additionally, two post-mission (PM) time points were measured on 4–7 (PM1), and day 13–14 (PM2) as shown in Table 2.

### Assessment of vascular stiffness

Rates of the movement of the pressure waves were used to assess pulse wave velocity (PWV) using Vicorder device. The device measures the velocity of waves transmitted between two points across the walls of the large carotid and femoral arteries. Operating in connection with a laptop software program, the device analyzes the waveform, delivering the speed measurement in meters per second (m/s). After a period of 20 min in a supine position to ensure relaxation, the assessment of PWV was undertaken. The detailed guidelines for using the Vicorder device have reported earlier (McGreevy et al., 2013).

### Non-invasive retinal vessel’s assessment

Retinal images were collected serially with a non-mydriatic hand-held retinal camera (Optomed, Finland) according to the study protocol. Trained graders from crew members have performed vessel measurements on the optic disc–centered image of the right eye. For detailed processing of retinal images see methods in Saloň et al. (2023). The image analysis was done using the IVAN software (University of Wisconsin, Madison, WI). This approach has previously been used in other population-based studies (Chandra et al., 2019).

### Ethics statement and informed consent

The study incorporated human participants and received thorough evaluation and endorsement from the Bioethical Commission at the Institute of Biomedical Problems of the Russian Academy of Sciences (Protocol No. 539 of 17 March 2020). The study fully adhered to the tenets outlined in the Declaration of Helsinki.

Each study participant voluntarily signed an informed consent after they were comprehensively briefed on the potential risks, advantages, and objectives of the forthcoming research.

## Results

The present study was conducted with a total of 5 adult participants. However, due to data artifacts in 1 participant, the analysis and presentation below only involved 4 participants. These participants had a mean age of 34.75 ± 5.44 years, height of 170.00 ± 2.00 cm, weight of 74.50 ± 12.53 kg, and average BMI of 25.47 ± 3.94 kg/m^2^, as shown in Table 1.

**TABLE 1:** Demographics of the four crew members completed the mission.

S. No	Sex	Age (years)	Height (cm)	Weight (kg)	BMI
CM-1	F	33	168	67	23.7
CM-2	F	30	172	67	22.6
CM-3	M	44	172	96	32.4
CM-4	M	32	168	68	24.1

CM, crew member; F, female; M, male; BMI, Body Mass Index (kg/m^2^).

### Retinal arteriolar and venular diameters

**FIGURE 1:**
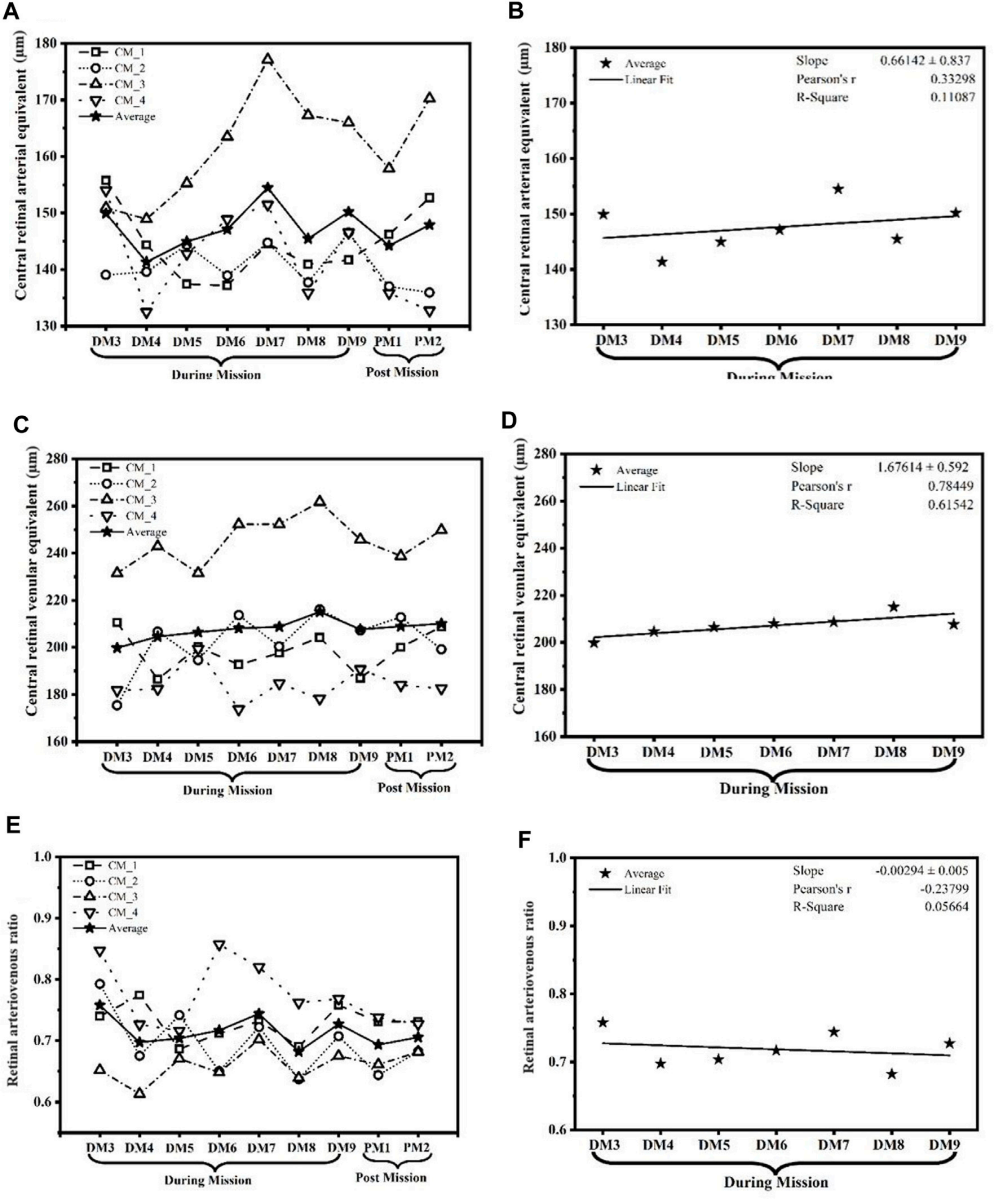
The graph showcases the temporal changes and potential tendencies in **(A,B)** central retinal arteriolar equivalent (CRAE) in µm; **(C,D)** central retinal venular equivalent (CRVE) in µm; and **(E,F)** arteriovenous ratio (AVR). These parameters were measured across the duration of the SIRIUS-21 mission, illustrating potential trends that signify the impact of isolation and confinement on retinal vasculature. (CM, Crew member; DM, During mission; PM, Post mission).

Figure 1A shows the Central Retinal Arteriolar Equivalent (CRAE) in seven time points during mission/isolation (DM) and two times points post mission/isolation (PM) for four crew members separately and their means measured. The mean of all CRAE (µm) time points shows an upward trend with a slope of 0.661 ± 0.83, Pearson’s r 0.332 and R-square 0.11 (Figure 1B). Similarly, the CRVE values (µm) are shown in Figure 1C during mission/isolation and post mission/isolation. Figure 1D shows a steeper upward elevation of CRVE with the slope of 1.676 ± 0.59, stronger correlation with Pearson’s r 0.784, and a higher R square 0.62 compared to CRAE.

### Retinal arteriovenous ratio

Figure 1E shows the AVR at all seven time points during the mission/isolation (DM) and two time points post mission/isolation (PM) for four crew members separately as well as their average. Figure 1F shows the average AVR across all time points, which exhibits a downward trend with a slope of −0.00294 ± 0.005. In addition, the figure shows Pearson’s r of–0.238 and R-square of 0.057 which indicate a weak negative correlation between time and AVR, which means that as time goes on, there is a slight decrease in AVR. The actual values for the AVR during- and post-mission/isolation as well as their average are in Table 2. We used linear regression analysis as is depicted in Table 3. It appears that only crew member # 2 showed a statistically significant decrease trend over time, while the other crew members and the average did not show statistically significant trends. The t-value for crew member #2 is −0.624, which is significant at a 5% significance level (*p*-value = 0.036).

**TABLE 2:** The arterio-venular ratio of the retinal images analysis in each crew member and their averages in various time points during and post mission/isolation.

Participant	DM3	DM4	DM5	DM 6	DM7	DM8	DM9	PM1	PM2
CM-1	0.739946	0.774305	0.6864	0.711838	0.731762	0.690208	0.75794	0.730906	0.731276
CM-2	0.792701	0.675404	0.741695	0.650148	0.722413	0.637196	0.707341	0.643787	0.682582
CM-3	0.65206	0.613222	0.670512	0.64823	0.702036	0.639206	0.675268	0.66115	0.681595
CM-4	0.847261	0.726646	0.716217	0.857117	0.820063	0.762225	0.768329	0.738282	0.727212
Average	0.757992	0.697394	0.703706	0.716833	0.744069	0.682209	0.727219	0.693531	0.705666

CM, crew member; DM, during mission; PM, post mission.

**TABLE 3:** Linear regression analysis of retinal images. Only CM_2 (crew member 2) shows a statistically significant decreasing trend over time (*p*-value = 0.0359), while the other crew members and the average do not show statistically significant trends.

Participant	Slope	Intercept	Correlation coefficient	T-value	*p*-value
CM-1	−0.000246904	0.7320885	−0.035427704	−0.06478998	0.949792461
CM-2	−0.002688155	0.7460516	−0.183576128	−0.62356398	*0.035865034
CM-3	0.001642172	0.6477883	0.057988743	0.188463818	0.856356767
CM-4	0.004338102	0.4583304	0.478190867	1.428220161	0.195829728
Average	0.000429559	0.7023972	0.122088976	0.469355064	0.649776546

### Assessment of vascular stiffness

**FIGURE 2:**
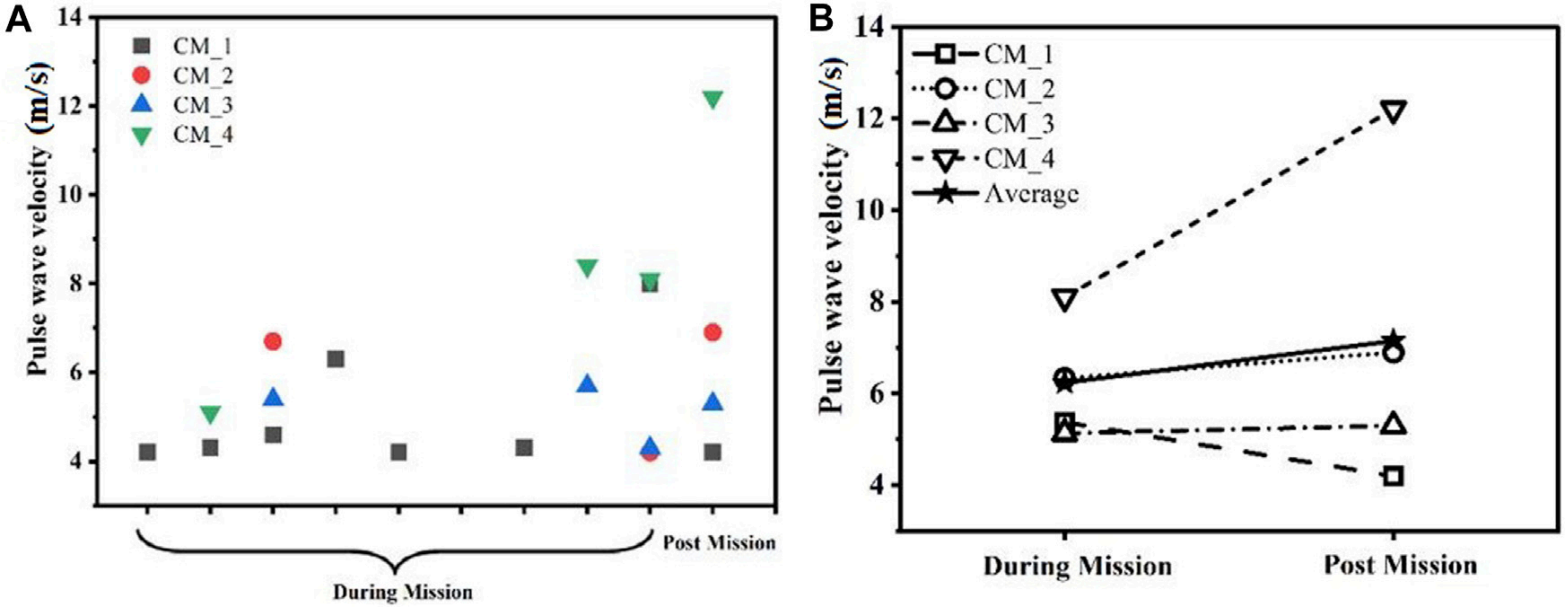
The graph showcases the **(A)** changes and **(B)** potential tendencies in pulse wave velocity (PWV). PWV (m/s) was measured across the duration of the SIRIUS-21 mission. (CM, Crew member; DM, During mission; PM, Post mission).

The arterial stiffness was measured using pulse wave velocity (PWV) of the four crew members and their average, as shown in Figure 2A, during mission/isolation. However, a few data were not counted in the study due to the presence of artifact in them. Thus, fewer measurements were included in calculation. Figure 2B shows the average of all PWV (m/s) time points with an upward trend with a positive linear relationship between independent variable during mission and post mission with intercept = −1.081, slope 0.914.

## Discussion

Our study focused on its effect of 8-month isolation and confinement on small retinal vessels as well as vascular stiffness of larger vessels. Our results indicate a decreasing trend (although not statistically significance) of the average retinal arteriolar-to-venular diameter ratio (AVR) due to retinal venular dilation during and post isolation, suggesting an increased risk of several health conditions, including cardiovascular disease, stroke, hypertension, and, potentially, dementia. Additionally, we observed an increasing trend (again not statistically significance) in pulse wave velocity, indicating a tendency toward vascular stiffness and a higher risk for cardiovascular diseases.

The vascular dysfunction tendency observed in our study is consistent with a previous study of isolation and confinement in the MARS-500 mission conducted in the same facility. Using echography in this study, Arbeille et al. (2014), have shown that the retinal vessel intima media thickness, but not the diameters, were significantly increased (14%–28%, *p* < 0.05) during isolation and immediately post-isolation in all six crew members. Monitoring changes in the retinal blood vessels is critical for the early detection and management of various cardiovascular and cerebrovascular disorders. An excellent meta-analysis has previously reported the predictive value of retinal vessel diameters for incident stroke in multiple large cohort studies (Streese et al., 2020). A follow-up period of 5–12 years documented a total of 945 stroke incidents (4.5%), revealing that wider venular diameters were associated with a higher incidence of stroke. The data indicated that for every 20 μm increase in venular diameter, the risk of stroke increased by 15%, with no significant correlation to arteriolar diameter, according to the Rotterdam Scan Study (McGeechan et al., 2009). This study concluded that venular dilation warrants further attention, as it could provide new insights into the pathophysiology of cerebral small vessel disease, echoing the suggestions made by our findings. Relevant to the limited physical activity in the isolated and confined conditions experienced by our participants, a wider CRVE (Central Retinal Vein Equivalent) was observed in sedentary individuals at risk compared to healthy ones, a finding supported by several other studies that described the impact of exercise on retinal vessel diameters (Hanssen et al., 2011; Streese et al., 2019). Consequently, an optimized and enhanced physical exercise program may be necessary in isolated environments during prolonged spaceflight. Additionally, CRVE widening has been associated with inflammatory states related to obesity, diabetes, and dyslipidemia, all of which may correlate with our findings but require further future investigations (Ikram et al., 2004; Liu et al., 2021). Although challenging to fully explain, the tendency for CRAE dilation rather than narrowing, potentially induced by stress in our isolated environment, is notable. This is especially significant since previous reports have linked CRAE narrowing with hypertension and endothelial dysfunction, both precursors to atherosclerosis and increased cardiovascular disease risk (Chew et al., 2012). For instance, a 16-year follow-up study among several others provided evidence that CRAE narrowing and CRVE widening were associated with a higher incidence of heart failure in both men and women (Chandra et al., 2019). The exact relationships between various retinal vascular parameters (CRAE, CRVE, and AVR) and the increased risk of cardiovascular disorders are not fully understood and required further investigations. However, in line with our findings, a decrease in the Arteriovenous Ratio (AVR) has consistently been observed in the majority of studies as a useful parameter for stratifying cardiovascular risk in patients (French et al., 2022).

Several cellular and molecular mechanisms have been shown to result in changes in retinal arteriolar and venular caliber (CRAE and CRVE) and arteriolar-to-venular ratio (AVR), especially during aging, and increased cardiovascular risk. These mechanisms include increased endothelin levels, reactive oxygen species, inflammation, insulin resistance, visceral adiposity, blood pressure, as well as decreased nitric oxide (NO) and other vasodilators, and endothelial progenitor cells (EPCs) (Wong et al., 2001; Gu et al., 2016; Hanssen et al., 2022).

Arterial stiffness has been shown to occur after a 6-month stay on the International Space Station. In both male and female astronauts, post-flight data showed up to 17%–30% changes in β-stiffness index (*p* = 0.006) as compared to preflight data (Hughson et al., 2016). Beside the effect of microgravity on inducing arterial stiffness in this study, a portion of this stiffness could be due to the stress induced by isolation and confinement, which would be consistent with the trend in our study that needs to be verified further in future and larger studies.

The limitation of our study is the smaller sample size, which is a common challenge in space-related studies. Therefore, further studies involving more participants are needed to confirm our results and achieve statistical significance, and to possibly elucidate the underlying mechanisms. In addition, some artifact data points were noticed due to, most likely, errors and unexperienced crew members in conducting the measurements. Thus, we considered only the reliable data points to draw most accurate conclusion.

Taken together, our results suggest that during 8-month isolation period of the SIRIUS analog mission, there is a trend of lower AVR and increased CRVE, which predicts a higher tendency for crew members to experience cerebrovascular and cardiovascular events in the future. The steeper upward elevation of CRVE with the slope of 1.676 ± 0.59, stronger correlation with Pearson’s r 0.784 compared to CRAE. These results revealed a stronger connection between CRVE and time points relative to the CRAE. This aspect of our conclusion should be interpreted with caution until further studies can verify it. Furthermore, our findings indicate a potential increase in vascular stiffness during and after the 8-month isolation period. This trend of higher arterial stiffness at the end of the mission may also place these analog participants at higher risk of cardiovascular diseases. Overall, our findings underscore the importance of investigating the effects of isolation and confinement on vasculature and highlight the need for continued research in this area as we work towards the goal of manned missions to Mars and beyond.

## Data Availability

The original contributions presented in the study are included in the article/Supplementary material, further inquiries can be directed to the corresponding author.
